# Supplementary material for: Liver X Receptor Agonist Inhibits Oxidized Low-Density Lipoprotein Induced Choroidal Neovascularization via the NF-κB Signaling Pathway
Source: J Clin Med. 2023 Feb 20;12(4):1674. doi: 10.3390/jcm12041674 (PMC9964355; doi:10.3390/jcm12041674)
Supplement: Supplementary file 1 [file jcm-12-01674-s001.zip › jcm-2100620-supplementary.pdf]

Journal of
Clinical Medicine

MDPI

Supplementary Materials

Table S1. Primary antibodies used in western blotting.

Antibody Name	Host Species	Concentrations	Catalog Number	Company
β-actin	Mouse	1:2000	3700	CST, American
GAPDH	Rabbit	1:2000	5174	CST, American
Histone H3	Mouse	1:1000	3638	CST, American
IL-1β	Rabbit	1:1000	ab9722	Abcam, American
IL-6	Rabbit	1:1000	ab6672	Abcam, American
CCR2	Rabbit	1:1000	ab203128	Abcam, American
VEGF	Rabbit	1:1000	ab46154	Abcam, American
TNFα	Mouse	1:1000	60291-1-Ig	Proteintech, China
ABCA1	Mouse	1:1000	ab18180	Abcam, American
ABCG1	Rabbit	1:1000	ab52617	Abcam, American
LXR	Goat	1:1000	ab24362	Abcam, American
CD31	Rabbit	1:1000	ab28364	Abcam, American
NF-κB p65	Rabbit	1:1000	8242	CST, American
IκB	Mouse	1:1000	4814	CST, American
p- IκB	Rabbit	1:1000	2859	CST, American
AKT	Rabbit	1:1000	4691	CST, American
p-AKT	Rabbit	1:1000	4060	CST, American

Table S2. Sequence of primers for mouse.

Gene	Forward Primer	Reverse Primer
β-actin	AAC AGT CCG CCT AGA AGC AC	CGT TGA CAT CCG TAA AGA CC
IL-6	TAGTCCTTCCTACCCCAATTTCC	TTGGTCCTTAGCCACTCCTTC
CCR2	ATCCACGGCATACTATCAACATC	CAAGGCTCACCATCATCGTAG
VEGF	GCACATAGAGAGAATGAGCTTCC	CTCCGCTCTGAACAAGGCT

Table S3. Sequence of primers for human.

Gene	Forward Primer	Reverse Primer
β-actin	CTCCATCCTGGCCTCGCTGT	GCTGTCACCTTCACCGTTCC
LXR	AGAAGATTCGGAAACAACAGCA	GCTGGATCATTAGTTCTTGAGCC

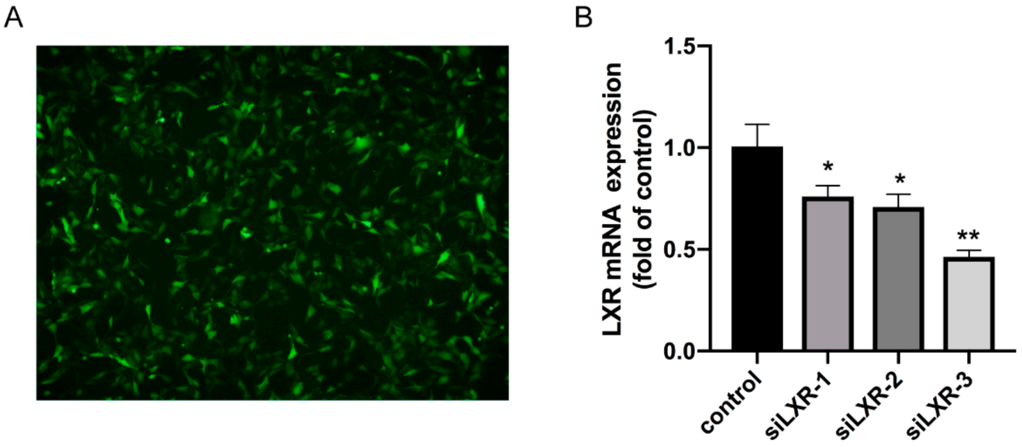

Figure S1. siRNA transfection efficiency (A) Cell transfer siLXR-GFP efficiency (B) LXR mRNA ex-pression, n=3, $^{*}P< 0.05$, $^{**}P< 0.01$, $^{***}P< 0.001$ vs. control group.
